# Comparing time to recovery in wasting treatment: simplified approach vs. standard protocol among children aged 6–59 months in Ethiopia—a cluster-randomized, controlled, non-inferiority trial

**DOI:** 10.3389/fped.2024.1337370

**Published:** 2024-05-22

**Authors:** Yetayesh Maru, Dessalegn Tamiru, Kaleab Baye, Stanley Chitekwe, Yehenew G. Kifle, Arnaud Lailou, Tefera Belachew

**Affiliations:** ^1^ Nurition and Dietetics Department, Faculty of Public Health, Jimma University, Jimma, Ethiopia; ^2^ Center for Food Science and Nutrition, Addis Ababa University, Addis Ababa, Ethiopia; ^3^ Nutrition and Food Systems Division, Research Center for Inclusive Development in Africa (RIDA), Addis Ababa, Ethiopia; ^4^ Nutrition Section, UNICEF, Addis Ababa, Ethiopia; ^5^ Department of Mathematics and Statistics, University of Maryland Baltimore County, Baltimore, MD, United States; ^6^ Nutrition Section, UNICEF West and Central Africa Regional Office, Dakar, Senegal

**Keywords:** moderate acute malnutrition, nutritional need, recovery, severe acute malnutrition, simplified approach, wasting

## Abstract

**Introduction:**

Wasting occurs when the body's nutritional needs are unmet due to insufficient intake or illness. It represents a significant global challenge, with approximately 45 million infants and children under 5 years of age suffering from wasting in 2022.

**Methods:**

A cluster-randomized, controlled, non-inferiority trial was conducted in three regions of Ethiopia. A non-inferiority margin of 15%, along with a recovery rate of 90% and a minimum acceptable recovery rate of 75%, were considered alongside an intra-cluster correlation coefficient of 0.05 and an anticipated loss to follow-up of 10% in determining the total sample size of 1,052 children. Children with severe acute malnutrition (SAM) in the simplified group received two sachets of ready-to-use therapeutic food (RUTF) daily, while the standard group received RUTF based on their body weight. For moderate acute malnutrition (MAM) cases, the simplified group received one sachet of RUTF, whereas the standard group received one sachet of ready-to-use supplementary food daily. A non-parametric Kaplan–Meir curve was utilized to compare the survival time to recovery.

**Results:**

A total of 1,032 data points were gathered. For SAM cases, the average length of stay was 8.86 (±3.91) weeks for the simplified protocol and 8.26 (±4.18) weeks for the standard protocol (*P* = 0.13). For MAM cases, the average length of stay was 8.18 (±2.96) weeks for the simplified approach and 8.32 (±3.55) weeks for the standard protocol (*P* = 0.61). There was no significant difference (*P* = 0.502) observed between the simplified protocol [8 weeks, interquartile range (IQR): 7.06–8.94] and the standard protocol [9 weeks (IQR: 8.17–9.83)] among children with SAM on the median time to cure. There was no significant difference (*P* = 0.502) in the time to cure between the simplified approach [8 weeks (IQR: 7.53–8.47)] and the standard protocol [8 weeks (IQR: 7.66–8.34)] among children with MAM. The survival curves displayed similarity, with the log-rank test not showing significance (*P* > 0.5), indicating the non-inferiority of the simplified approach for cure time.

**Conclusion:**

The findings showed that the simplified and standard protocols demonstrated no significant differences in terms of the average duration of stay and time required for recovery.

**Clinical Trial Registration:**

https://pactr.samrc.ac.za/, Identifier (PACTR202202496481398).

## 1 Introduction

Wasting occurs when the body's nutritional needs are not met because of insufficient intake or disease (1). In children under 5 years of age, wasting is defined as having a weight-for-height or weight-for-length *z*-score (WLZ) less than 2 standard deviations (SD) below the median of the World Health Organization (WHO) child growth standards [weight-for-height z-score (WHZ) or WLZ < −2] or a mid-upper-arm circumference (MUAC) less than 125 mm. The global burden of wasting is substantial, with approximately 45 million infants and children under 5 years of age experiencing wasting in 2022, and efforts to reduce its prevalence have shown limited progress. Wasting and nutritional edema have severe consequences, including immediate susceptibility to disease and death. Surviving infants and children often face challenges in motor and cognitive development, persisting into adulthood and resulting in reduced economic productivity and a higher risk of non-communicable diseases (2).

Interventions aimed at treating wasting and nutritional edema have significantly contributed to enhancing child survival and meeting international goals (3–6). Over the past two decades, community management approaches for acute malnutrition have demonstrated that children with uncomplicated severe acute malnutrition (SAM) and moderate acute malnutrition (MAM) can be effectively managed at the outpatient community level, incurring lower costs compared with inpatient care (7). In recent years, there has been a shift toward a simplified approach to the treatment of acute malnutrition, aiming to enhance coverage and reduce expenses. This streamlined approach emphasizes the use of a single product [ready-to-use therapeutic food (RUTF)] for managing severe and moderate acute malnutrition (8–14). In addition, it includes the utilization of MUAC measurements less than 125 mm or the presence of edema as admission criteria (15–35). Moreover, for the treatment of children with severe acute malnutrition, reduced or modified dosages of RUTF have been incorporated into the approach (8, 13, 36–38).

In studies conducted in various regions of Africa, the duration of treatment and recovery time for children with acute malnutrition using a modified dosage was similar to the standard protocol (8–11, 14, 39). In this study, we set out to compare the recovery time of a simplified approach with the standard protocol in the treatment of acute malnutrition (specifically uncomplicated SAM and MAM in children aged 6–59 months) to provide context-specific evidence for treatment effectiveness.

## 2 Methods and materials

### 2.1 Study designs and settings

A cluster-randomized, controlled, non-inferiority trial with two-arm parallel design was conducted in three regions of Ethiopia: (1) Oromia; (2) Southern Nations, Nationalities, and Peoples’ Region (SNNPR); and (3) Amhara. These regions accounted for nearly two-thirds of the total SAM admissions nationwide (40).

**Figure 1:**
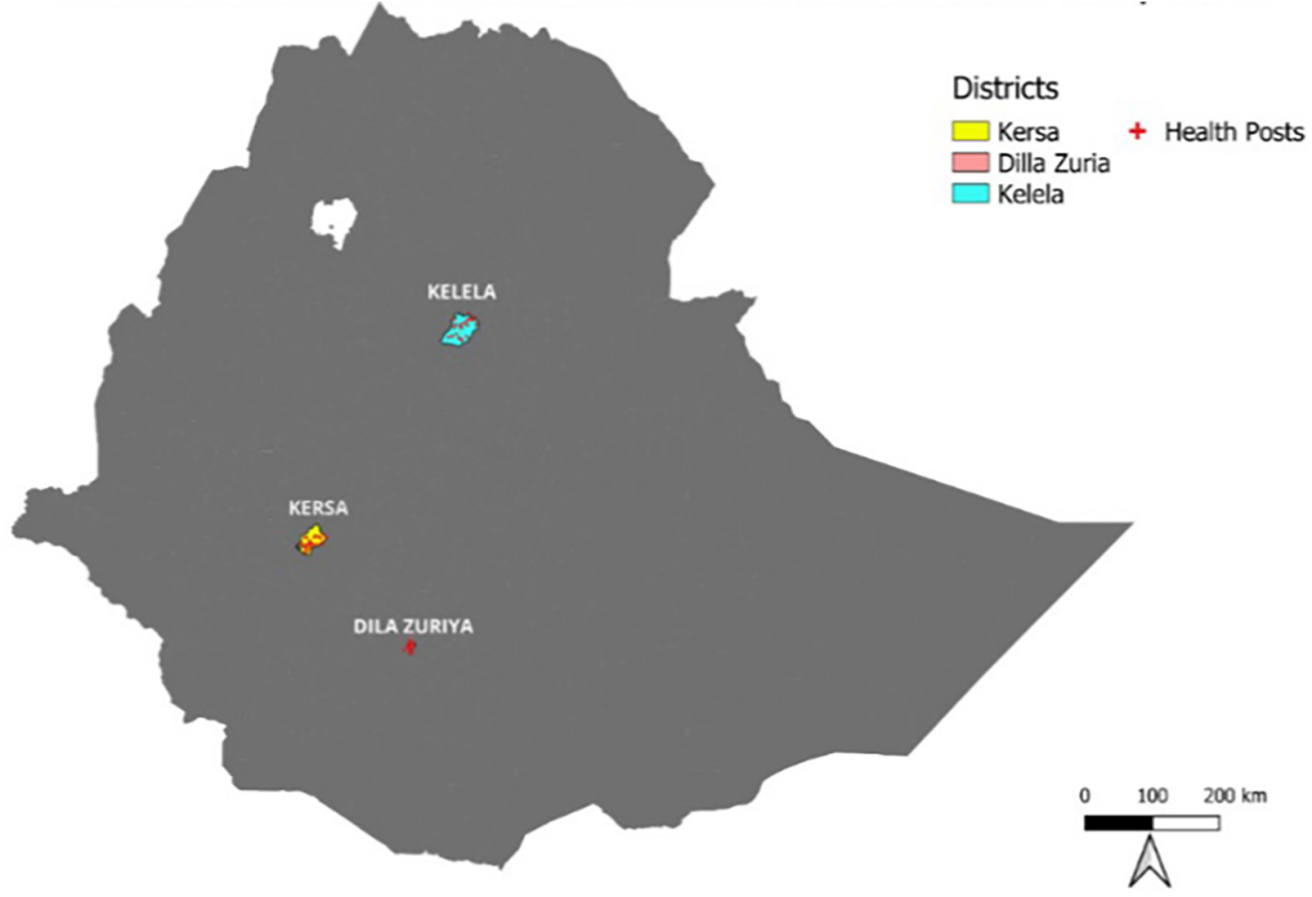
Map of the study area.

Data collection occurred from 4 December to 30 July 2022, involving the selection of *woredas*, based on their SAM caseload. The woredas are districts, which are the third level of administrative divisions in Ethiopia after region and zone. Consequently, Kersa Woreda in Jimma Zone (Oromia), Dilla Zuria Woreda (SNNPR), and Kelela Woreda (Amhara) were included in the trial (Figure 1). Kersa Woreda, situated in a rural area, has a total population of 178,035, including 36,814 children under the age of 5. Dilla Zuria Woreda has a population of 106,042, including 20,241 children under the age of 5, and Kelela Woreda has a total population of 144,576, including 22,407 children under the age of 5. Across these three woredas, there are a combined total of 93 health posts (41).

The context of the three regions selected for the study includes a high proportion of SAM admissions (38.4% in Oromia, 12.8% in Amhara, and 11.9% in SNNPR) (UNICEF 2019) and high wasting prevalence (15% in Amhara, 10% in SNNPR, and 9% in Oromia). There was also low coverage of nutrition services such as Vitamin A supplementation (21% in Oromia, 16% in Amhara, and 15% in SNNPR), deworming of children (29% in Oromia, 15% in Amhara, and 15% in SNNPR), and age-appropriate Infant and Young Child Feeding (IYCF) counseling (25% in Oromia, 33% in Amhara, and 25% in SNNPR) (EPHI 2022). The percentage of children who received all basic vaccines was 62.9% in Amhara, 43.5% in SNNPR, and 29.2% in Oromia (EPHI 2019).

Clusters were health posts that provided service to an average of 5,000 people in Ethiopia's rural settings. A total of 58 clusters were randomized in each woreda into Intervention (*n* = 29) and Control (*n* = 29), with an average of 18 children per cluster. The sample size was calculated using GPower 3.1 to detect a non-inferiority margin of 15% in recovery rates between the simplified protocol and the standard protocol. A recovery rate of 90% was assumed, based on previous program data in the country, and a minimum acceptable recovery rate of 75% per Sphere Standards was considered in the worst-case scenario (42).

Taking into account an intra-cluster correlation coefficient of 0.05 (43) and a 10% loss to follow-up yielded a total sample size of 1,052 children (430 SAM and 622 MAM). A total of 58 health posts (clusters) were estimated using an estimation formula for the number of clusters (*n*) = intra-cluster correlation (0.05) × the sample size (1,052) and adding 10% for non-response. The sample size was also calculated for subgroup analysis by the type of wasting, as follows: For Children with SAM, a sample size of 430 (215 Intervention, 215 Control) was estimated assuming a 95% confidence interval, power of 87%, precision of 5%, an allocation ratio of 1, and a design effect of 1.85 calculated using inter-cluster correlation (ICC) of 0.05 and cluster size of 18. Similarly, for children with MAM, a sample size of 622 (311 Intervention, 311 Control) was estimated assuming a 95% confidence interval, power of 90%, a precision of 5%, an allocation ratio of 1, and an effect of 1.85 calculated using ICC of 0.05 and cluster size of 18.

The study enrolled children based on specific eligibility criteria, which included the following: uncomplicated SAM with MUAC less than 125 mm or bilateral pitting edema of Grade+or ++, WHZ below −3 or between −3 and −2, passing appetite test, absence of medical complications, and residence in the designated catchment areas at the time of inclusion. Children were excluded from the study if they met any of the following criteria: (i) severe anemia, defined as a hemoglobin concentration less than 4 g/dl; (ii) severe dehydration; (iii) hypothermia or active infection; (iv) plans to relocate from the catchment area within the next 6 months; or (v) presence of malformations or disabilities such as cleft palate, cerebral palsy, or Down's syndrome that could impact food intake.

### 2.2 Randomization and blinding

**Figure 2:**
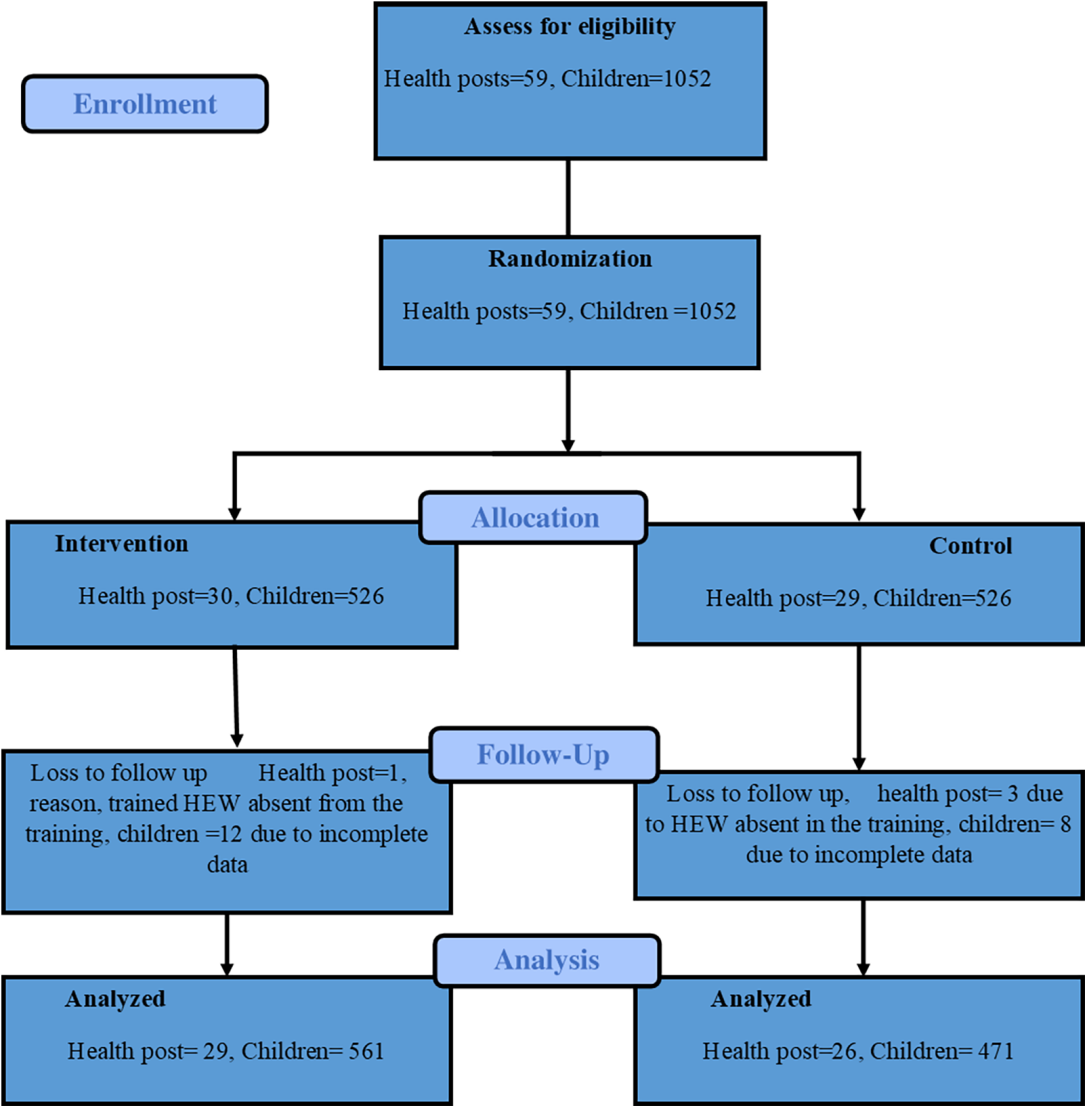
CONSORT flow diagram of the cluster-randomized trial (43).

Clusters (health posts) were selected from the functional outpatient therapeutic program (OTP) sites and that admitted a minimum of two children monthly. A comprehensive list of eligible health posts served as the sampling frame. All 58 eligible health posts (clusters) were listed according to their stratification by woreda, and ENA SMART software was used to generate a random number of health posts for the intervention and control groups (ENA 2020). The eligible 58 health posts (clusters) were stratified into the respective woredas and randomized into Intervention and Control arms using ENA SMART software (Figure 2). Children exhibiting MUAC less than 12.5 cm and/or bilateral edema of Grade+or ++ were admitted for acute malnutrition treatment. Children with wasting and complications, such as severe illness, danger signs, or a failure to pass the appetite test, were referred to stabilization centers (SC) for further evaluation and care. Given the nature of distributing treatment supplies, blinding the study participants was not feasible. However, blinding procedures were implemented for data collectors and the data monitoring supervisor.

#### 2.2.1 Measurement Methods

A mother–child electronic scale was used to measure weight. Weight was recorded to the nearest 100 g (0.1 kg). A standard weight of 1 kg was used for daily calibration of the scale. MUAC was measured with a non-stretchable MUAC tape on the left arm to the nearest mm. Length and height were measured to the nearest 0.1 cm with a wooden height board with graduated index strips in millimeters on each side. Children less than 87.0 cm or 24 months of age were measured lying down, and children with height greater or equal to 87.0 cm or 24 months were measured in the standing position. The presence of nutritional edema was determined by applying moderate pressure for about 3 s. If there is edema, an impression remains for some time (at least a few seconds) where the edema fluid is pressed out of the tissue. The child can only be recorded as edematous if both feet have edema. The same procedure was repeated at the lower legs to check for the degree of edema.

Regular clinical assessment of each child was conducted on a weekly basis (i.e., for temperature, respiratory rate, pulse rate, cough, ear, and nasal discharges) according to the national protocol for the management of SAM. Symptoms, diagnoses, and treatments prescribed were recorded. The clinical assessment also served to monitor the development of complications that need inpatient care. All clinical complications were immediately reported to the study supervisor, and the child was referred to inpatient treatment if needed.

### 2.3 Implementation procedures

A training program spanning five days was conducted for 30 technical participants at each administrative level. The purpose was to impart knowledge to these participants, enabling them to subsequently train health extension workers (HEWs) on both simplified and standard protocols. The objective was to ensure consistent implementation of these protocols. Following this, a 4-day training session was organized for the HEWs from 58 health posts out of a total of 59. In total, 149 HEWs and health workers were trained, with 67 from standard (Control) health posts and cluster health centers, and 82 from simplified (Intervention) health posts and health centers within the catchment areas. The training sessions were structured to introduce the simplified protocol to the Intervention clusters initially, and subsequently, the standard protocol was taught to the Control clusters in the second round. To aid the process, a simplified approach guide, specifically designed for managing uncomplicated acute malnutrition at the health post level, was created in the local language. Meanwhile, the existing reference materials available for HEWs were used for the standard protocol. Various tools, including registration books, admission cards, home visit forms, and death report forms, were prepared to facilitate the implementation and monitoring of the trial in both groups.

Children diagnosed with acute malnutrition based on criteria such as MUAC measurement of <125 mm or the presence of Grade+or Grade ++ edema were admitted to either the simplified or standard groups, depending on the randomly selected health posts. Notably, the WHZ criterion was not applied at the health post level since WHZ measurements are exclusively conducted at the health center and hospital levels. Children with an MUAC measurement of <115 mm or Grade+or ++ edema were categorized as severe acute malnutrition, and those without medical complications were admitted to OTP at the health post level.

### 2.4 Intervention

*Simplified approach*: Implementing a unified program for both SAM and MAM using RUTF. Children with SAM received two RUTF sachets daily, while patients with MAM were given one RUTF sachet daily. The *standard approach* treated SAM with RUTF and MAM with ready-to-use supplementary food (RUSF). RUTF dosage was adjusted based on the weights of the children with SAM. The amount of RUTF was provided based on a child's weight by referring to the quick reference table prepared based on the national guideline for managing acute malnutrition. The amount of RUTF increased when the child's weight increased, whereas MAM cases were provided with one sachet of RUSF.

The nutritional composition of RUTF in 100 g is 520–550 kcal, with 13–17 g of protein; 26–37 g of lipids; essential vitamins such as Vitamin A, B, C, and E; and minerals such as iron and zinc. Similarly, the nutrition composition of RUSF in 100 g is 510–560 kcal, with 11–16 g protein; 26–36 g lipids; essential vitamins such as Vitamin A, B, C, and E; and minerals such as iron and zinc.

In both groups, measurements of MUAC, weight, presence of edema, and body temperature were taken during each visit. Height measurements were recorded upon admission and discharge. In addition, a clinical examination was conducted once the child showed no improvement in MUAC, had a fever, or exhibited other health issues. Children diagnosed with SAM in both groups were administered standard medications: (i) amoxicillin upon admission at a dosage of 25 mg/kg every 12 h for 5 days, (ii) albendazole as a single oral dose of 400 mg on the second visit for children older than 24 months, and (iii) measles vaccine during the fourth week of the visit for children aged 9–59 months, provided that they hadn't received the vaccine previously.

Children with MUAC measurements ranging from ≥115 to <125 mm were categorized as having MAM and were treated at the health post level, receiving treatment similar to that of SAM, but at the same location. In the Intervention (simplified) group, children with MAM received one sachet of RUTF daily with a follow-up schedule every fortnight. Conversely, children in the standard group were given one sachet of RUSF per day during their bi-weekly visits. Furthermore, children older than 24 months with MAM in both the simplified and standard groups received albendazole as a single 400 mg dose upon admission. Children aged 9–59 months with MAM were vaccinated against measles upon admission if they had not received the vaccination previously.

Both the standard and simplified approaches used the same discharge criteria. Children diagnosed with SAM were deemed cured and discharged when they met the criteria of having MUAC >125 mm for two consecutive weekly visits, displayed good clinical health, and had a minimum stay of 3 weeks. Children admitted based on edema were discharged when there was an absence of bilateral pitting edema for two consecutive weekly visits and when the child was clinically well and alert, which applied to both the Intervention (simplified) and standard groups. Similarly, children with MAM were considered cured and discharged when their MUAC was ≥125 mm for two consecutive measurements taken every 2 weeks. In addition, they needed to be clinically well and have a minimum stay of three weeks, a criterion applied uniformly to both the intervention (simplified) and standard groups. Children with SAM and MAM in both the intervention (simplified) and control groups were classified as defaulters if they were absent for two consecutive visits. A non-responder status was confirmed when they did not meet the discharge criteria even after 16 weeks (4 months) of treatment.

Regular anthropometric measurements were taken as referred in the measurement method. In cases where children lacked birth or vaccination certificates or any documented age, their ages were estimated using a local event calendar, which included significant festivals, construction events, and religious observances. Weekly clinical evaluations of the child included assessments of temperature, respiratory rate, pulse rate, cough, ear, and nasal discharges. These assessments adhered to the national protocol for managing SAM symptoms, diagnoses, and prescribed treatments, with outcomes meticulously documented. The clinical assessments also tracked the emergence of complications requiring inpatient care. Children exhibiting clinical complications were promptly referred for inpatient treatment.

Regular on-site monitoring was done to oversee the application of both the simplified and standard approaches for treating acute malnutrition. In each woreda, a nutrition officer was appointed to closely monitor the simplified/standard approach's implementation in the health posts. These officers ensured weekly home visits to children with SAM to monitor the proper administration of RUTF and routine medications. In addition, the women Health Development Army members were oriented to conduct daily home visits to meet children with SAM, ensuring they received the prescribed RUTF dosage and routine medication. They also educated mothers and family members about not sharing RUTF and made random checks on its usage. The Health Development Army members were equipped with a checklist, which they completed and submitted to HEWs weekly. Similarly, HEWs conducted weekly home visits to children with SAM in both the Intervention (simplified) and Control (standard) groups. Mothers were educated not to share RUTF/RUSF provided to children with uncomplicated SAM or MAM. Mothers were also instructed to retain the empty sachets of RUTF/RUSF and display them during Health Development Army visits and bring them to the health posts during their appointments.

### 2.5 Data collection procedures

Experienced data collectors were recruited based on their prior expertise in data collection. A comprehensive 3-day training was conducted to acquaint them with the latest research updates, review the questionnaires, and provide hands-on practical training on usage of the Open Data Kit (ODK) platform. The training covered various aspects, including the use of ODK, height and weight measurements, MUAC measurements, registration procedures, admission cards, and consent form protocols. The 19 data collectors selected from the three woredas were randomly assigned to specific health posts. Each data collector was responsible for collecting data from three to five health posts per week. The allocation of data collectors to health posts was organized based on the health posts’ OTP day schedules. Data collectors visited the health posts on their respective OTP days. To ensure effective communication, the questionnaire was initially developed in English and then translated into the local languages: Amharic for the Kelela and Dila Zuria woredas, and Oromiffa for Kersa Woreda. The translation quality was assessed through a thorough review of each question with the data collectors.

Data were collected using ODK. At admission, mothers/caregivers were interviewed individually, about areas such as the following: (i) socio-economic status, encompassing demographic information, wealth index, household water sanitation, and hygiene conditions; (ii) infant and young children, focusing on breastfeeding and complementary feeding practices for children aged 6–23 months; and (iii) nutrition services, including child participation in the Growth Monitoring and Promotion Program, Vitamin A supplementation program, deworming initiatives, and common morbidities.

Prior to the actual data collection, the questionnaire underwent rigorous pretesting, and necessary modifications were made based on the feedback received. To maintain quality, the data were meticulously reviewed by supervisors, data managers, and the principal investigator to ensure completeness and consistency. This rigorous oversight and the thorough training procedures were implemented to ensure the accuracy and reliability of the collected data.

### 2.6 Data analysis

An intention-to-treat analysis (ITT) was conducted to compare the simplified group and standard group. The data were extracted from ODK and imported into SPSS version 20 for thorough cleaning and analysis. Recoding was performed for variables such as the type of acute malnutrition, treatment outcomes, and recovery status.

To visualize the time taken for cure across treatment groups, a non-parametric Kaplan–Meier curve was employed. This curve displays the survival (time-to-cure) among the treatment groups. A log-rank test was performed to compare the median survival time among these two groups. Further analysis involved isolating factors independently associated with the time taken to cure. This was achieved using a multivariable Cox proportional hazard (PH) model and an advanced clustered frailty model, which accounted for the clustering effect present in the data. Hazard ratios and adjusted hazard ratios, accompanied by 95% confidence intervals, were calculated. To ensure the reliability of the analysis, the statistical assumptions of proportional hazard were verified using SPSS version 20. Associations with a *P*-value less than 0.05 were considered statistically significant.

### 2.7 Ethical consideration

Ethical approval was granted by the Ministry of Science and Higher Education's National Research Ethics Review Committee in Ethiopia. In addition, a letter of support from the Ministry of Health was sent to all three study regions and woredas. The study strictly adhered to the principles outlined in the Helsinki Declaration. To ensure ethical compliance, written and well-informed consent was obtained from mothers/caregivers participating in the study. They were fully aware of their right to withdraw from the trial at any point.

Before obtaining consent, data collectors thoroughly explained the informed consent form to potential participants. Participants were given ample opportunity to ask questions before deciding to voluntarily participate in the study. All questionnaires and consent forms were translated into Amharic/Oromiffa prior to the commencement of the study. To maintain confidentiality, the information collected was kept secure and was not shared with others, especially if it contained personally identifiable information.

The trial was registered on Pan African Clinical Trial Registry with a unique registration number (PACTR202202496481398).

## 3 Results

Complete data were collected from a total of 1,032 children aged 6–59 months suffering from uncomplicated severe acute and moderate acute malnutrition. When considering the duration of their stay in the treatment program, there was no significant difference observed between the simplified and standard groups, whether for SAM or MAM cases. For SAM cases, the average length of stay was 8.86 (±3.91) weeks in the simplified group and 8.26 (±4.18) weeks in the standard group (*P* = 0.13). Similarly, in the case of MAM, the average length of stay was 8.18 (±2.96) weeks in the simplified group and 8.32 (±3.55) weeks in the standard group (*P* = 0.61) (Table 1).

**Table 1:** Comparing a simplified approach to the standard protocol in treating children with severe and moderate acute malnutrition in three regions of Ethiopia.

Type of acute malnutrition	Treatment approach	Sample size (*n*)	Length of stay (in weeks)		Difference	*P*
			Mean	SD	Mean (95% CI)	
SAM	Simplified	231	8.86	3.91	0.59 (−0.18 to 1.37)	*0* *.* *13*
	Standard	195	8.26	4.18		
MAM	Simplified	330	8.18	2.96	−0.14 (−0.66 to 0.38)	*0* *.* *61*
	Standard	276	8.32	3.55		

Std, standard deviation; SAM, sever acute malnutrition; MAM, moderate acute malnutrition.

The italicized *p*-value above 0.05 in Table 1 shows that there is no statistically significant difference in the survival curve (time to recovery) between the two groups (Simplified versus Standard) for both SAM and MAM groups of children.

Regarding the median time to recovery for children with severe acute malnutrition, no statistically significant difference was observed (*P* = 0.502) between the standard protocol, which took 8 weeks [interquartile range (IQR): 7.06–8.94], and the simplified protocol, which took 9 weeks (IQR: 8.17–9.83). Similarly, there was no significant disparity (*P* = 0.224) in the time to recovery among children with MAM between the standard protocol, which took 8 weeks (IQR: 7.53–8.47), and the simplified approach, which took 8 weeks (IQR: 7.66–8.34) (Table 2).

**Table 2:** Comparing a simplified approach and standard protocol in treating children with SAM and MAM: median time to cure (in weeks) in three regions of Ethiopia.

Type of acute malnutrition	Treatment approach	Median (IQR)	*P* ^a^
SAM	Standard	8.00 (7.06 to 8.94)	0.502
	Simplified	9.00 (8.17 to 9.83)	
	Overall	9.00 (8.38 to 9.62)	
MAM	Standard	8.00 (7.53 to 8.47)	0.224
	Simplified	8.00 (7.66 to 8.34)	
	Overall	8.00 (7.65 to 8.35)	

^a^ *p*-values are based on log-rank test statistic.

**Figure 3:**
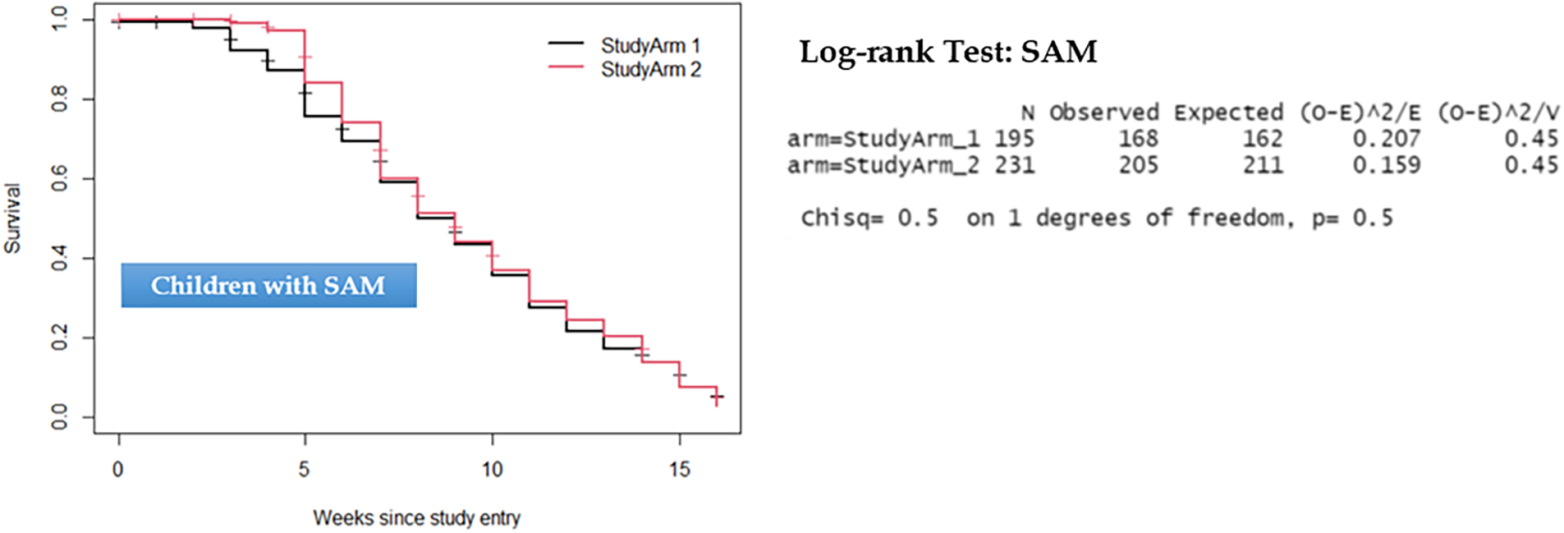
Kaplan–Meier survival curve and log-rank test results comparing recovery time for children with severe acute malnutrition.

**Figure 4:**
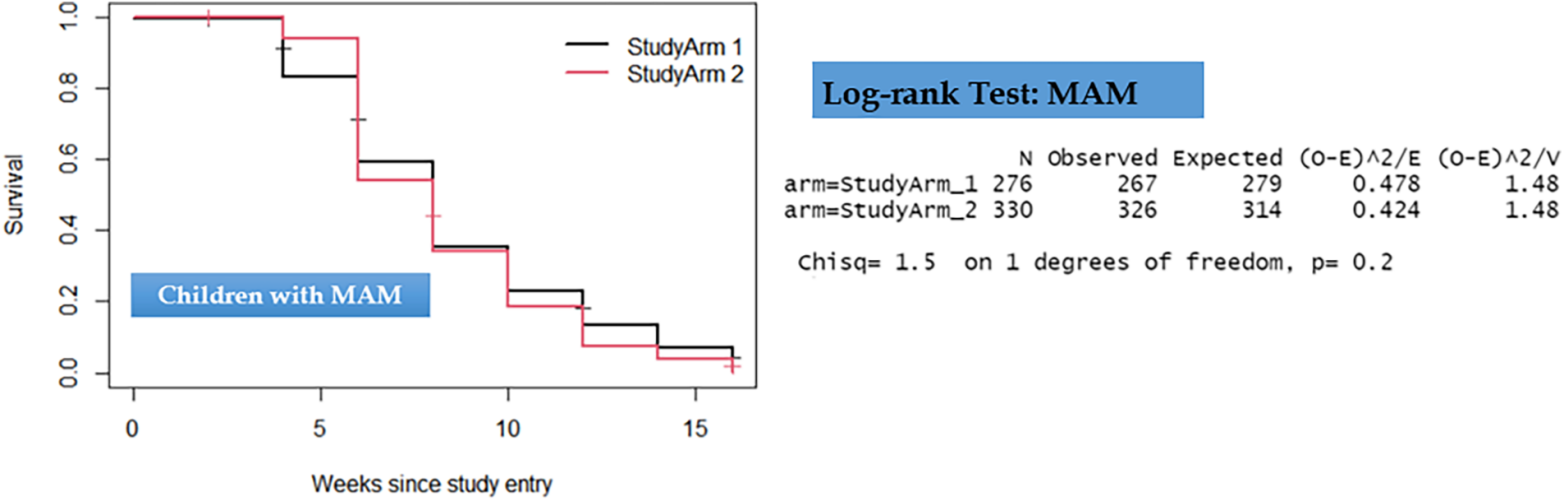
Kaplan–Meier survival curve and log-rank test results comparing recovery time for children with moderate acute malnutrition.

Comparison of the two groups in terms of recovery time showed that there was no statistically significant difference observed among children with uncomplicated SAM and those with MAM. As depicted in Figures 3, 4, the survival curves exhibited similarity, and the log-rank test yielded non-significant results (*P* > 0.5), signifying that the simplified approach was non-inferior in terms of recovery time for both SAM and MAM.

In addition to the log-rank test, we applied a clustered Cox PH model, also known as a shared frailty model, to both SAM and MAM datasets. Like the results from the log-rank tests, the outcomes from the shared frailty models for both SAM and MAM datasets revealed non-significant differences between the two treatment groups concerning recovery time (*P* = 0.58 and *P* = 0.61), respectively. Moreover, significant clustering or heterogeneity was observed in both SAM and MAM groups, indicating the appropriateness of using clustered survival models for these datasets (*P* < 0.01). This approach not only validated the results but also enhanced the precision of the model parameters, as presented in Table 3.

**Table 3:** Comparing treatment arms using shared frailty model for children with SAM and MAM.

Type of acute malnutrition	Factors	Levels	Coef	SE (Coef)	HR	Chisq	*P*
SAM	Study arm	Arm 1 (Ref)					
		Arm 2	−0.078	0.139	0.925	0.314	0.580
	Frailty (cluster)						
						39.43	*0* *.* *0094*
MAM	Study arm	Arm 1 (Ref)					
		Arm 2	0.078	0.154	1.081	0.256	0.610
	Frailty (cluster)						
						115.390	*<0*.*0001*

SE, standard error; Chisq, chi square.

The italicized *p*-value above 0.05 in Table 3 shows that there is no statistically significant difference in the MEDIAN survival time (MEDIAN time to recovery) between the two groups (Simplified versus standard) for both SAM and MAM groups of children.

As depicted in Table 4, the non-significant difference in the median time to recovery remained consistent even after adjusting for various background variables (*P* = 0.14 for SAM and *P* = 0.58 for MAM). Only two variables were significantly associated with the time to recovery for children with SAM while none of the background variables were significantly associated with time to cure for children with MAM. For the children with SAM, paternal education of primary and above [adjusted hazard ratio (HR): 1.44, *P* = 0.02] and age at baseline greater that 24 months (adjusted HR: 1.56, *P* = 0.03) were significantly associated. However, for both SAM and MAM, there was a significant clustering or heterogeneity, which is captured by the clustered survival models.

**Table 4:** Multivariable shared frailty model predicting recovery time for children with SAM and MAM in Ethiopia.

Factors	Levels	SAM				MAM			
		Coef	SE(Coef)	Hazard ratio	*P*-value	Coef	SE(Coef)	Hazard ratio	*P*-value
Study arm	Arm 1 (Ref)								
	Arm 2	–0.2592	0.1739	0.7717	0.14	0.0891	0.1631	1.0932	0.58
Sex	Male (Ref)								
	Female	0.1356	0.1312	1.1453	0.30	0.0372	0.0878	1.0379	0.67
Mother's education	No education (Ref)								
	Primary and above	–0.3039	0.1754	0.7380	0.08	0.0534	0.1038	1.0549	0.61
Mother's occupation	Have job (Ref)								
	Housewife	–0.0090	0.1497	0.9911	0.95	0.0299	0.1155	1.0304	0.80
Paternal education	No education (Ref)								
	Primary and above	0.3646	0.1669	1.4400	0.03	0.0353	0.0997	1.0359	0.72
Paternal occupation	Other job (Ref)								
	Farmer	–0.4586	0.2412	0.6322	0.06	0.0755	0.1310	1.0784	0.56
Family size (count)		0.0380	0.0371	1.0387	0.31	0.0392	0.0247	1.0400	0.11
Age at baseline	<24 Months (Ref)								
	≥24 Months	0.4425	0.1938	1.5566	0.02	0.0301	0.1034	1.0305	0.77
MDDS	Inadequate (Ref)								
	Adequate	0.2529	0.2846	1.2878	0.37	0.0856	0.1738	1.0893	0.62
Waste disposal	Unsafe (Ref)								
	Safe	–0.0250	0.1541	0.9753	0.87	–0.0105	0.1091	0.9896	0.92
Vitamin A Supp	No (Ref)								
	Yes	0.0949	0.1635	1.0996	0.56	0.0720	0.1218	1.0746	0.55
Wealth index (count)		–0.0085	0.0995	0.9916	0.93	0.0082	0.0622	1.0082	0.90
Frailty (cluster)		0.011				<0.0001			

MDDS, The minimum dietary diversity score.

## 4 Discussion

We found no significant differences in the average length of stay for children with SAM and MAM between the simplified and standard treatment protocols. For SAM, the average length of stay was 8.86 weeks in the simplified group compared with the 8.26 weeks in the standard group (*P* = 0.13). Likewise, for MAM, the average length of stay was 8.18 weeks in the simplified group and 8.32 weeks in the standard group (*P* = 0.61). The median time to cure also showed no significant difference; children with SAM took 8 weeks to recover in the standard group (IQR: 7.06–8.94) and 9 weeks in the simplified group (IQR: 8.17–9.83) protocols (*P* = 0.502), while children with MAM recovered in 8 weeks from both standard (IQR: 7.53–8.47) and simplified (IQR: 7.66–8.34) protocols (*P* = 0.224). Children with uneducated parents and those younger than 24 months of age had a longer SAM recovery time than those older and from educated parents. However, such relationships were not observed for children with MAM. Furthermore, survival curves’ analyses and the non-significant log-rank test (*P* > 0.5) demonstrated the non-inferiority of the simplified approach in terms of cure time compared with the standard group. This similarity persisted even after employing the shared gamma frailty model (*P* = 0.575), indicating clustering or heterogeneity in both SAM and MAM groups. These findings are consistent with previous studies conducted in various regions of Africa, supporting the consistency of non-inferiority in average length of stay and time to cure between simplified and standard treatment approaches (8–11, 14, 39). These findings altogether have strong programmatic and policy implications. First, the finding that SAM and MAM treatments can be optimized to be delivered through the same product (RUTF) and unified protocol may have significant savings in resources relative to the current approach that treats MAM and SAM separately using different protocols, products, and institutional setups. Second, the mainstreaming of MAM treatment will enable earlier identification and treatment of children in the wasting process, leading to fewer hospitalization and inpatient care visits (43). Third, such savings can help fill the significant coverage gap, with less than 25% of children with SAM being currently treated (44).

The strength of our study was that it covered three large areas of Ethiopia, consisting of about two-thirds of the national acute malnutrition caseload, and used the existing structure of community-based interventions through close monitoring at health posts and via home visits. Moreover, our study applied the Kaplan–Meir curve to display the survival (time to cure) among treatment groups after adjusting for different background characteristics of the children and the factors associated with time to cure isolated using the Cox proportional hazard model with advanced clustered frailty model to capture the clustering effect. The limitation is that the study doesn't address acute emergency contexts.

The findings of this study further strengthened the simplified and combined treatment approach guideline developed for emergency setup by the federal ministry of health in Ethiopia (45). Given both the global and national shortage of supplies for the treatment of acute malnutrition, the findings imply the need for looking into strategies for wider application of the simplified approach in the routine non-emergency setups. However, the performance of simplified approached on time to recovery in the emergency context needs to be evaluated.

## 5 Conclusion

Our findings support the conclusion that the simplified treatment protocol, which does not significantly differ from the standard protocol in terms of average length of stay and time to recovery, is a viable approach for managing acute malnutrition in children. These results underline the effectiveness of the simplified approach and its potential for widespread implementation in similar contexts and suggest that a transition to a simplified approach would result in a non-inferior outcome.

## Data Availability

The raw data supporting the conclusions of this article will be made available by the authors, without undue reservation.
